# Upregulation of E2F8 promotes cell proliferation and tumorigenicity in breast cancer by modulating G1/S phase transition

**DOI:** 10.18632/oncotarget.8121

**Published:** 2016-03-16

**Authors:** Liping Ye, Ling Guo, Zhenyu He, Xi Wang, Chuyong Lin, Xin Zhang, Shu Wu, Yong Bao, Qi Yang, Libing Song, Huanxin Lin

**Affiliations:** ^1^ Department of Experimental Research, State Key Laboratory of Oncology in Southern China, Collaborative Innovation Center for Cancer Medicine, Sun Yat-sen University Cancer Center, Guangzhou 510060, People's Republic of China; ^2^ Department of Nasopharyngeal Carcinoma, State Key Laboratory of Oncology in Southern China, Collaborative Innovation Center for Cancer Medicine, Sun Yat-sen University Cancer Center, Guangzhou 510060, People's Republic of China; ^3^ Department of Radiation Oncology, State Key Laboratory of Oncology in Southern China, Collaborative Innovation Center for Cancer Medicine, Sun Yat-sen University Cancer Center, Guangzhou 510060, People's Republic of China; ^4^ Department of Breast Surgery, State Key Laboratory of Oncology in Southern China, Collaborative Innovation Center for Cancer Medicine, Sun Yat-sen University Cancer Center, Guangzhou 510060, People's Republic of China

**Keywords:** E2F8, proliferation, tumorigenicity, breast cancer, cell cycle

## Abstract

E2F transcription factors are involved in cell cycle regulation and synthesis of DNA in mammalian cells, and simultaneously play important roles in the development and progression of cancer when dysregulated. E2F8, a novel identified E2F family member, was found to be associated with the progression of several human cancers; however, the biological role and clinical significance of E2F8 in breast cancer remain to be further elucidated. Herein, we report that E2F8 is robustly elevated in breast cancer cell lines and clinical breast cancer tissue samples, respectively. The high expression level of E2F8 significantly correlates with clinical progression (*P* = 0.001), poor patient survival (*P* < 0.001) and a high Ki67 staining index (*P* = 0.008) in 187 human breast cancer specimens. Furthermore, we find that overexpressing E2F8 promotes, whereas silencing E2F8 suppresses, the proliferation and tumorigenicity of breast cancer cells both *in vitro* and *in vivo*. We further demonstrate that E2F8 transcriptionally upregulates CCNE1 and CCNE2 via directly interacting with their respective gene promoter, which accelerates the transition of G1 to S phase of breast cancer cells. Taken together, these findings uncover a novel biologic role and regulatory mechanism of E2F8 responsible for the progression of breast cancer, indicating E2F8 may represent a novel prognostic biomarker and therapeutic target against breast cancer.

## INTRODUCTION

Sustaining proliferation is thought to be the most fundamental hallmark of cancer [1]. Generally, the production and release of proliferative signals that instruct entry into cell cycle progression are dysregulated in cancer cells, thereby breaking homeostasis of cell number and causing uncontrolled cell proliferation. During the cell cycle progression, G1/S phase transition emerges as one of the most critical steps, which involves activation of cyclin-dependent kinases (CDKs) by forming cyclins-CDK complex, phosphorylation of retinoblastoma (Rb), and E2F-mediated gene transcription [2]. For instance, cyclin E (including cyclin E1 and cyclin E2) binds to CDK2, which in turn phosphorylates Rb to promote G1/S phase progression. Consistently, dysregulation of cyclin E-CDK2 activity is involved in various types of cancers, including breast, gastric, kidney and lung cancer, contributing to uncontrolled cell proliferation [3–7]. In fact, cyclin E is frequently amplified in breast cancer, and cyclin E overexpression is associated with a poor clinical benefit of breast cancer, while inhibition of cyclin E-CDK2 activity dramatically reduces proliferation and tumor formation and considered as a therapeutic approach in cancer [8–12]. Therefore, more detailed knowledge of cell cycle transition mechanisms would not only be beneficial to understanding the initiation and progression of breast cancer, but may also provide new clues for the development of novel therapeutic strategies.

The E2Fs (E2F1-E2F8) are a large family of transcription factors containing one or more conserved DNA binding domains (DBDs) that bind to target promoters and regulate their expressions [13]. E2F family transcription factors are critical for many developmental processes, and regulate cell cycle and DNA synthesis in mammalian cells. Notably, dysregulation of E2F proteins contribute to cancer initiation and progression. It was reported that ablation of E2F1, E2F3 or E2F4 in Rb^+/−^ mice significantly suppressed the development of pituitary tumors, extending the tumor-free lifespan of Rb1^+/−^ mice [14–16]. In contrast, loss of E2F2 increased Myc-induced T cell lymphomagenesis in mice, and the reintroduction of E2F2 into E2F2-null tumors resulted in apoptosis of the tumor cells [17]. Moreover, E2F1-5, but not E2F6 or E2F7, are elevated and correlate with a higher proliferation index and a poorer clinical outcome in breast cancer [18–22]. Hence, these findings provide substantial evidence to demonstrate that E2Fs might function as a tumor suppressor or an oncogene during the progression and development of cancer. It has been reported that E2F8, in combination with E2F7, is required for embryonic development in mice [23, 24] and also angiogenesis [25] and lymphangiogenesis [26] in zebrafish. Interestingly, recent advances indicated that E2F8 is deregulated in several human cancers. Parisi et al. reported that the gene copy number of E2F8 was frequently gained in human melanoma [27]. Moreover, E2F8 has also been found to be overexpressed in ovarian cancer, hepatocellular carcinoma and lung cancer [28–30]. Reimer et al. found that overexpression of E2F8 was associated with histopathologic progression in ovarian cancer [28]. In addition, E2F8 promoted cancer cell proliferation, chemoresitance and invasion, and constituted a potential therapeutic target in hepatocellular carcinoma [29] and lung cancer [30]. Hence, these findings have provided substantial evidence that E2F8 may play a vital role in the malignant progression of cancer. However, the clinical significance and biological role of E2F8 during the progression of breast cancer remain to be elucidated.

In the present study, we show that E2F8 is markedly upregulated in human breast cancer and closely correlates with the clinicopathological features and prognosis of breast cancer. Overexpressing E2F8 dramatically promoted, whereas silencing E2F8 inhibited the proliferation and tumorigenicity of breast cancer cells both *in vitro* and *in vivo*. Furthermore, we demonstrate that E2F8 can promote entry into the G1/S phase of the cell cycle via transcriptionally upregulating cyclin E1 and cyclin E2 expression, thus contributing to cell proliferation and tumorigenicity in human breast cancer. Taken together, our findings indicate that E2F8 plays an important role in the progression of human breast cancer and suggest that E2F8 may be a potential target for human breast cancer treatment.

## RESULTS

### E2F8 is upregulated in breast cancer cell lines and tissues

**Figure 1:**
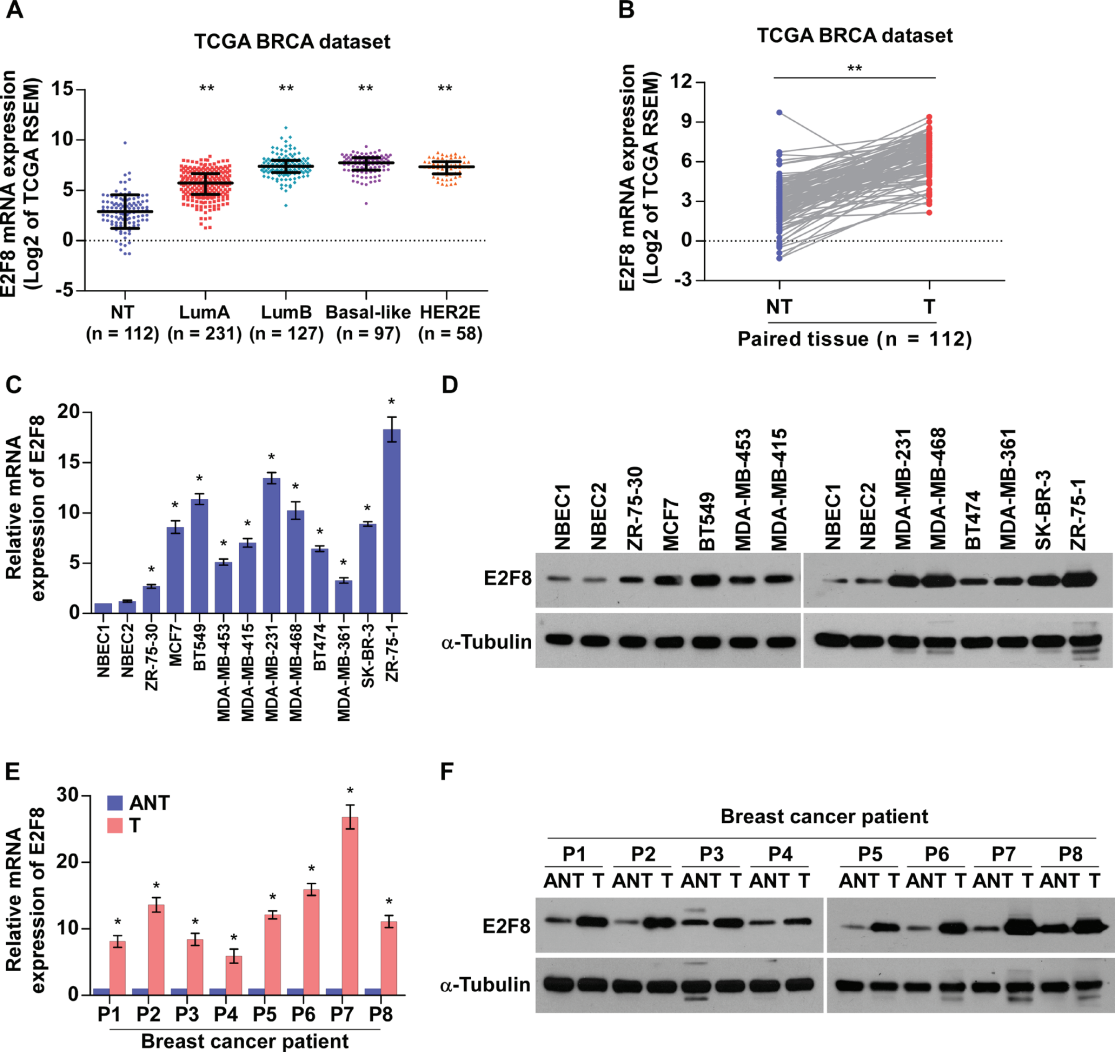
E2F8 is overexpressed in breast cancer cell lines and tissues (**A** and **B**) E2F8 mRNA levels in breast cancer tissues were assessed by analyzing TCGA breast cancer mRNA data set (A) (NT (non-tumor), *n* = 112; LumA (luminal A), *n* = 231; LumB (luminal B), *n* = 127; Basal-like, *n* = 97; HER2E (Her2-enriched), *n* = 58) and (B) the 112 paired adjacent non-tumor tissues (NT) and breast cancer tissues (T). Data were acquired from the TCGA data portal (https://tcga-data.nci.nih.gov/tcga/tcgaHome2.jsp). Lines represent mean ± SD. **P < 0.001, *t-test*. (**C** and **D**) Real-time PCR (C) and Western blotting (D) analyses detecting the mRNA and protein levels of E2F8 expression in two preparations of normal human breast epithelial cells (NBEC1 and NBEC2) and cultured breast cancer cell lines. (**E** and **F**) Real-time PCR (E) and Western blotting (F) analyses of E2F8 mRNA and protein expression in paired primary breast cancer tissues (T) and the matched adjacent non-tumor tissues (ANT) from eight breast cancer patients (P1-P8); mRNA expression was normalized to GAPDH and α-tubulin was used as a protein loading control. Data are mean ± SD of three independent experiments; *P < 0.05.

To investigate the clinical significance and biological role of E2F8 in breast cancer, we first analyzed the mRNA expression of E2F8 in breast cancer tissues with different molecular subtypes which show significant heterogeneity of breast cancer using published data from The Cancer Genome Atlas (TCGA) [31]. As shown in Figure 1A, E2F8 levels remained low in non-tumor breast tissues but became markedly higher in patients with luminal A and further elevated in other subtypes including luminal B, Basal-like and Her2-enriched, suggesting that E2F8 might contribute to high proliferation rates in breast cancer. Moreover, TCGA data analysis revealed that E2F8 levels were significantly upregulated in breast cancer tissues compared to paired tumor-adjacent non-tumor tissues (Figure 1B). Furthermore, we verified E2F8 expression in breast cancer cell lines and fresh tissues. Real-time PCR and western blotting revealed that E2F8, at both the mRNA and protein levels, was markedly overexpressed in all 11 tested breast cancer cell lines than that in normal breast epithelial cells (NBEC1 and NBEC2) (Figure 1C and 1D). Similarly, the mRNA and protein levels of E2F8 were differentially upregulated in all 8 freshly-frozen breast cancer samples as compared to the matched adjacent non-tumor tissues (Figure 1E and 1F), suggesting that E2F8 is upregulated in breast cancer cell lines and breast cancer tissues.

### Upregulation of E2F8 correlates with progression and poor prognosis in breast cancer

**Figure 2:**
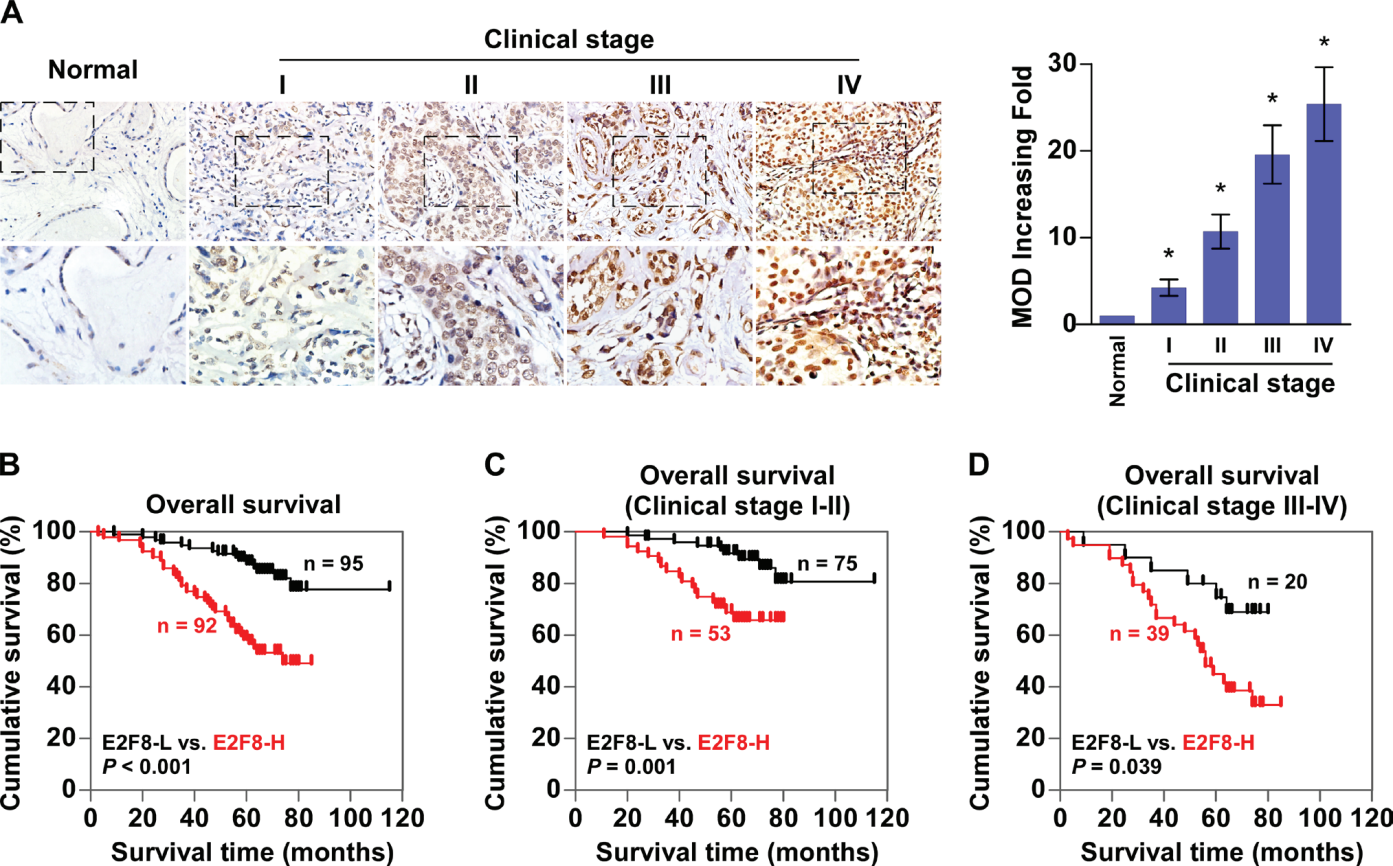
Upregulation of E2F8 correlates with progression and poor prognosis in breast cancer (**A**) Left panel: representative IHC analyses of E2F8 expression in normal breast tissue and breast cancer specimens of different clinical stages. Right panel: Statistical quantification of the mean optical density (MOD) of E2F8 staining between normal breast tissues and breast cancer specimens. *P < 0.05. (**B**) Kaplan-Meier analysis of overall survival curves for breast cancer patients with low E2F8 expression (E2F8-L; *n* = 95) versus high E2F8 expression (E2F8-H; *n* = 92) (*n* = 187; *P* < 0.001, log-rank test). (**C** and **D**) E2F8 expression also significantly correlated with overall survival in breast cancer patients with clinical stage 1 + 2 subgroup (*n* = 128; *P* = 0.001, log-rank test), or clinical stage 3 + 4 subgroup (*n* = 59; *P* = 0.039, log-rank test).

To evaluate whether E2F8 correlates clinically with breast cancer progression, the expression of E2F8 was examined by immunohistochemistry (IHC) in 187 paraffin-embedded, archived breast cancer tissues, including 33 cases of clinical stage I (17.6%), 95 cases of stage II (50.8%), 51 cases of stage III (27.3%) and 8 cases of stage IV breast cancers (4.3%) (Supplementary Table 1). Quantitative IHC analysis as determined by the mean optical density (MOD) showed that E2F8 expression increased along with disease stage in breast cancer (*P* < 0.05, Figure 2A). In agreement with this observation, χ^2^ test revealed that E2F8 levels significantly correlated with the clinical stage, and TNM classifications in patients with breast cancer (all *P* < 0.05) (Supplementary Table 2), indicating a positive correlation between E2F8 expression and breast cancer progression.

Furthermore, Kaplan-Meier and log-rank tests for survival analysis revealed that patients with high E2F8 expression had a significantly poorer overall survival compared to patients with low E2F8 expression (*P* < 0.001; Figure 2B). Notably, E2F8 expression also significantly correlated with overall survival in breast cancer patients with clinical stage 1 + 2 subgroup (*n* = 128, *P* = 0.001; Figure 2C), as well as clinical stage 3 + 4 subgroup (*n* = 59, *P* = 0.039; Figure 2D), suggesting that E2F8 might be a valuable prognostic marker for breast cancer patients at all disease stages. Interestingly, assessment from a publicly available breast cancer microarray data KM plotter [32] has shown a significant correlation between high expression of E2F8 and poor overall survival, relapse-free survival and distant metastasis-free survival of breast cancer patients (Supplementary Figure 1). Univariate and multivariate analyses indicated that clinical stage and expression of E2F8 and Ki67 were independent prognostic factors (Supplementary Table 3), which further supported the notion that E2F8 expression might represent a novel prognostic biomarker for the disease.

### Upregulation of E2F8 promotes proliferation of breast cancer cells

**Figure 3:**
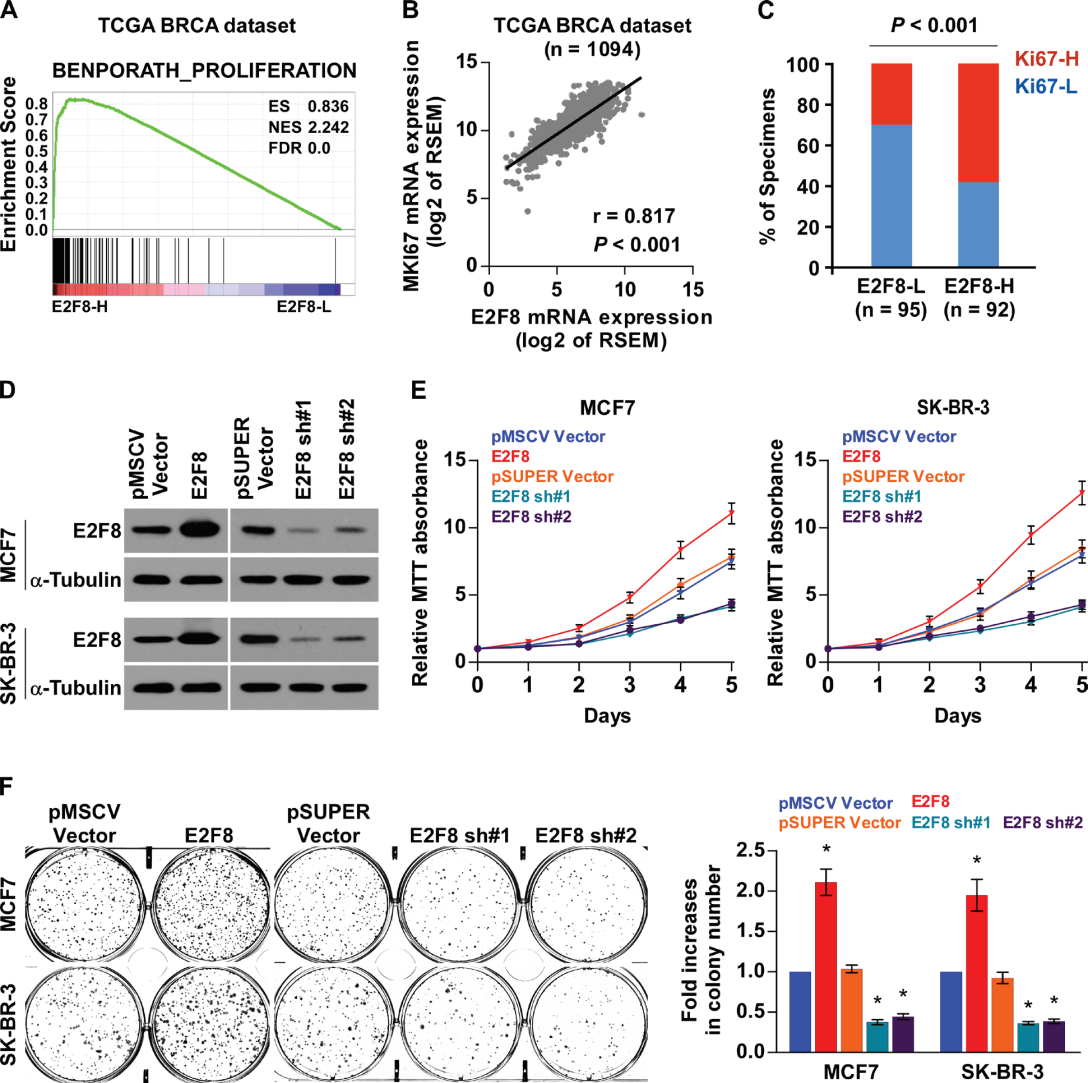
Upregulation of E2F8 promotes proliferation of breast cancer cells (**A**) GSEA plot performed using GSEA v2.2.0 (http://www.broadinstitute.org/gsea/) showed that high E2F8 expression was positively correlated with the cell cycle-associated gene signature (BENPORATH_PROLIFERATION) based on TCGA BRCA mRNA data set. (**B**) Correlations between E2F8 and MKI67 mRNA expression based on TCGA BRCA mRNA data set. r, Pearson correlation coefficient; *P* < 0.001. (**C**) The protein expression levels of E2F8 were associated with the expression of Ki67 in 187 primary breast cancer specimens. Percentage of specimens showing low or high Ki67 expression (Ki67-L and Ki67-H) in 187 primary human breast cancer specimens, related to the levels of E2F8. *P* < 0.001, χ^2^ test. (**D**) Western blotting analysis of E2F8 protein expression in the constructed MCF7 and SK-BR-3 cells; α-tubulin was used as a loading control. (**E**) Proliferation rate of the indicated breast cancer cells, as determined using the MTT assay. (**F**) Representative images (left panel) and quantification (right panel) of crystal violet-stained colony formation for the indicated cell lines. Data are mean ± SD of three independent experiments. *P <0.05.

The biological role of E2F8 in breast cancer was further explored using Gene Set Enrichment Analysis (GSEA) [33] based on mRNA expression data from the TCGA, which indicated that high levels of E2F8 correlated significantly with proliferation-associated gene signature (Figure 3A). Moreover, E2F8 expression levels were positively correlated with Ki67 expression from both TCGA mRNA data set (*r* = 0.817, *P* < 0.001) and our IHC results (*P* < 0.001) (Figure 3B, 3C), suggesting that E2F8 may contribute to cell proliferation in breast cancer.

We then evaluated the role of E2F8 in breast cancer cell proliferation by stably exogenously overexpressing, or endogenously knocking down of E2F8 expression via retrovirus infection (Figure 3D). An MTT assay showed that overexpression of E2F8 increased, while depletion of E2F8 expression reduced proliferation rates of both MCF7 and SK-BR-3 breast cancer cell lines (Figure 3E). Similar results were obtained in the colony formation assay (Figure 3F). Taken together, these data suggest that E2F8 plays important roles to promote breast cancer cell proliferation and colony formation *in vitro*.

### Upregulation of E2F8 enhances tumorigenicity of breast cancer cells

**Figure 4:**
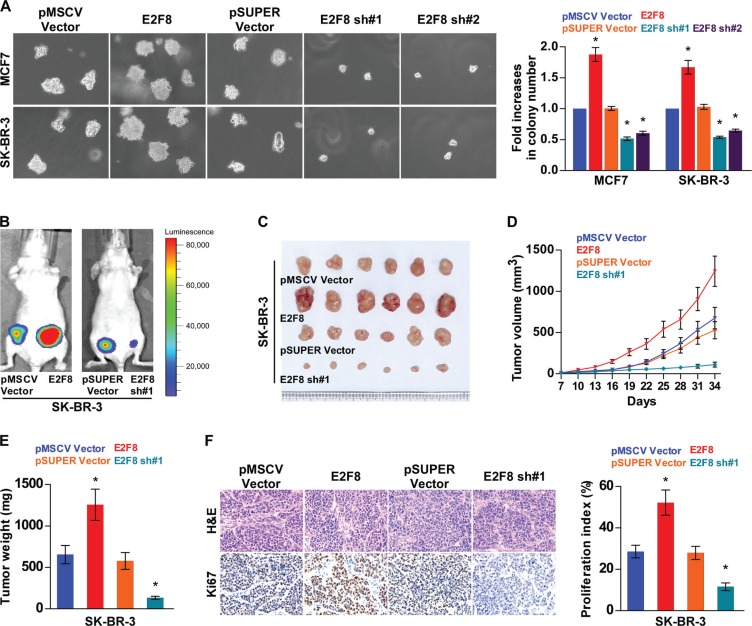
Upregulation of E2F8 enhances breast cancer cell tumorigenicity (**A**) Representative micrographs (left panel) and quantification (right panel) of colonies determined by anchorage-independent growth assay. Colonies larger than 0.1 mm in diameter were scored. (**B**) Xenograft model in nude mice. Representative images of tumor-bearing mice (B) and tumors (**C**) from each experimental group. (**D**) Volumes of tumors in the E2F8-overexpressing, E2F8-silencing, and control groups were measured on indicated days. Data presented are the mean ± SD. (**E**) Tumor weights of each group. (**F**) Proliferation index (right) was determined using the percentage of Ki67-positive cells (left). Data are mean ± SD of three independent experiments. *P < 0.05.

Since E2F8 expression was correlated with the clinical staging and TNM classification of breast cancer (Supplementary Table 2), we further evaluated the effect of E2F8 on the tumorigenic activity of breast cancer cells. First, we found that the anchorage-independent growth abilities of both MCF7 and SK-BR-3 breast cancer cell lines were significantly increased by overexpressing E2F8, but reduced by silencing E2F8 (Figure 4A). Furthermore, the transwell assay showed that silencing of E2F8 dramatically reduced the invasion capability of basal-like breast cancer cells MDA-MB-231 and BT549 (Supplementary Figure 2), suggesting that E2F8 expression played an important role in the invasive phenotypes of basal-like breast cancer cells.

Moreover, the role of E2F8 in the tumorigenicity of breast cancer cells was further determined *in vivo*. As shown in Figure 4B–4E, the E2F8-overexpressing tumors grew at a much higher rate in terms of size and weight, than the control tumors, whereas the tumors formed by E2F8-silenced cells were smaller and had lower tumor weights than the tumors formed from shRNA-vector control cells. Expression levels of E2F8 in xenografts were further examined by western blotting. E2F8 was robustly upregulated in tumors formed by SK-BR-3/E2F8 cells, but downregulated in tumors formed by E2F8-silencing SK-BR-3 cells (Supplementary Figure 3). Furthermore, IHC analysis revealed that E2F8-overepressing tumors displayed higher Ki67 proliferation index, whereas E2F8-silenced tumors showed reduced numbers of Ki67 positive cells (Figure 4F). Taken together, these results suggest that E2F8 promotes the tumorigenicity of breast cancer cells *in vivo*.

### E2F8 promotes the G1/S phase transition of breast cancer

**Figure 5:**
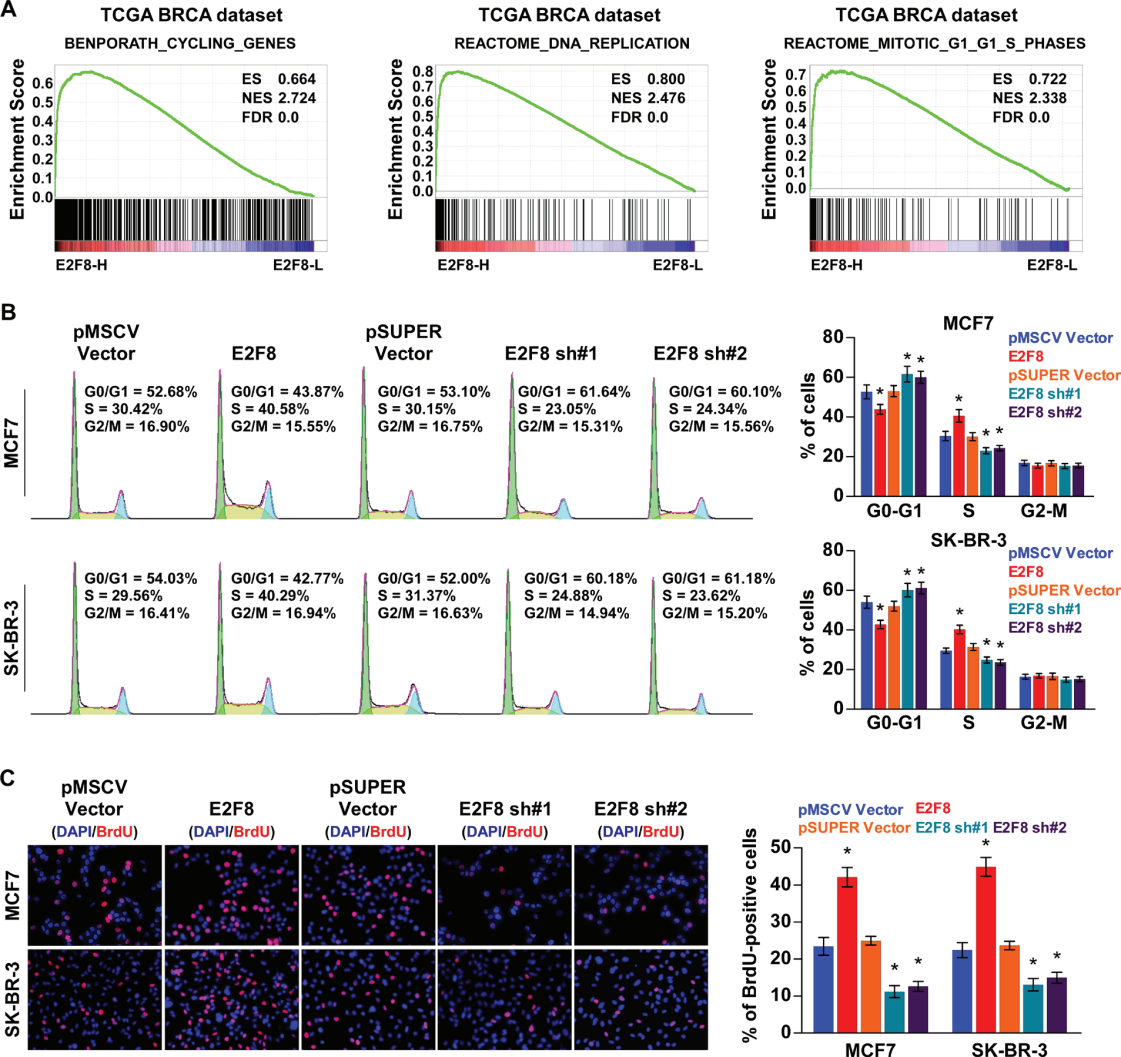
E2F8 promotes the cell cycle in breast cancer (**A**) GSEA results indicated that E2F8 expression was significantly correlated with the cell cycle-associated gene signatures including BENPORATH_CYCLING_GENES, REACTOME_DNA_REPLICATION, and REACTOME_MITOTIC_G1_G1_S_PHASES. (**B**) Flow cytometric analysis of indicated breast cancer cells. (**C**) Representative micrographs (left panel) and quantification (right panel) of BrdU incorporation in the indicated breast cancer cells. DAPI was used as a DNA/nuclear stain.

We then explored the mechanism underlying the promotion of cellular proliferation by E2F8. GSEA results indicated that high E2F8 expression was significantly correlated with the cell cycle-associated gene signatures, suggesting that E2F8 is involved in the cell cycle regulation (Figure 5A). Moreover, overexpression of E2F8 resulted in a significant increase in the percentages of cells in the S peak, but a decrease in the percentages of cells in the G0/G1 peak, whereas silencing E2F8 had the opposite effects (Figure 5B). Similarly, a BrdU incorporation assay revealed that the percentages of cells with incorporated BrdU was significantly enhanced in E2F8-overexpressing cells and reduced in E2F8-silenced cells (Figure 5C). Thus, these results indicate that E2F8 promotes cell cycle progression of breast cancer cells, confirming that E2F8 promotes breast cancer cell proliferation.

### E2F8 directly enhances the promoter activities of cyclin E1 and cyclin E2

**Figure 6:**
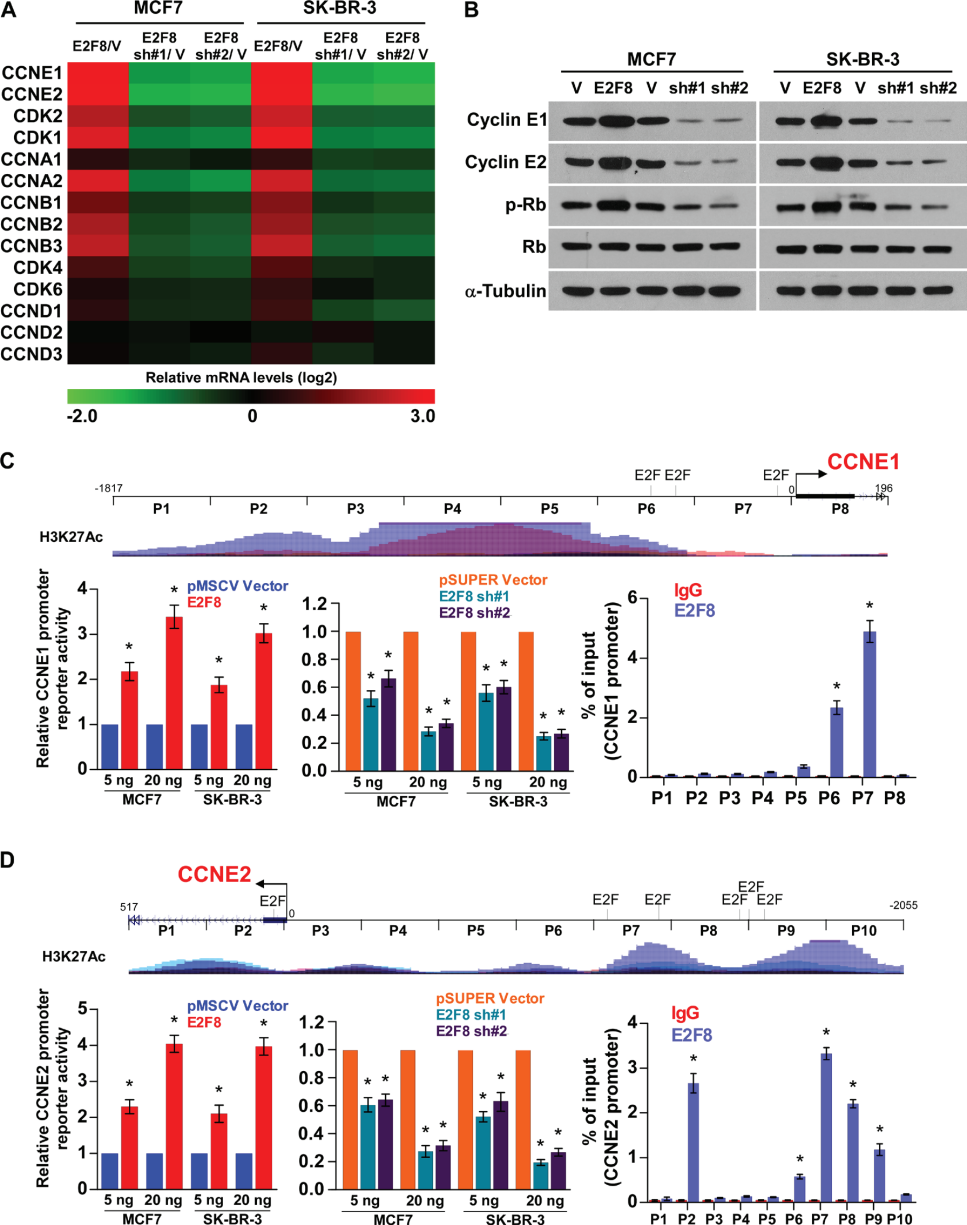
E2F8 directly upregulates the promoter activities of cyclin E1 and cyclin E2 (**A**) Real-time PCR analysis of cell cycle-related genes mRNA expression in the indicated cells. Gene expression levels were normalized to GAPDH. The pseudocolours represent the intensity scale of E2F8 versus pMSCV vector (V) or E2F8 sh#1/2 versus pSUPER vector (V), generated by log2 transformation. (**B**) Western blotting analysis of cyclin E1, cyclin E2, phosphorylated Rb (p-Rb) and total Rb protein expression in the indicated cells; α-tubulin was used as a loading control. (**C** and **D**) Upper panel: Schematic illustration of ChIP PCR fragments for the indicated nucleotide regions of the CCNE1 (C) and CCNE2 (D) promoters. Multiple typical response elements of E2F transcription factor were predicted using the ConSite program. H3K27Ac enrichment, indicating high transcription activity, is observed in the promoter elements according to Genome Browser Gateway website. Left panel: Luciferase activity assays in MCF7 and SK-BR-3 cells showed transactivation of the CCNE1 and CCNE2 promoters by E2F8 overexpression and repression by E2F8 silencing. Right panel: ChIP enrichment assay confirms that E2F8 binds to the predicted promoter site of CCNE1 and CCNE2; IgG was used as a negative control. Results were evaluated from three independent experiments, *P < 0.05.

Since E2F8 is involved in the cell cycle regulation of breast cancer cells, we examined the expression of cell cycle regulators. As shown in Figure 6A and 6B, real-time PCR and western blotting analysis revealed that multiple cell cycle regulators, especially CCNE1 and CCNE2, were robustly increased in E2F8-overexpressing cells, but reduced in E2F8-silenced cells compared to control cells. Consistent with this observation, correlation analysis in TCGA Breast Invasive Carcinoma (BRCA) data set reveals that E2F8 positively correlates with Cyclin E1 and Cyclin E2 (Supplementary Figure 4). Moreover, phosphorylation of Rb, the downstream target protein of cyclin E-CDK2 complex, was shown to be induced in E2F8-overexpressing cells, but suppressed in the E2F8-silenced cells (Figure 6B).

Interestingly, by analysis of the promoter regions of CCNE1 and CCNE2 using the ConSite [34] and Genome Browser Gateway website programs, we found multiple typical response elements of E2F transcription factor, suggesting that E2F8 might induce CCNE1 and CCNE2 expression by directly targeting their gene promoters. As expected, luciferase reporter assays revealed that overexpression of E2F8 activated, whereas downregulation of E2F8 attenuated, the luciferase activity of CCNE1 and CCNE2 promoters in breast cancer cells in a dose-dependent manner (Figure 6C, 6D). ChIP assays showed that E2F8 was capable of binding to different fragment regions within the CCNE1 and CCNE2 promoters (Figure 6C, 6D and Supplementary Figure 5A). To validate this, we performed ChIP assays by pulling downing transcription activation marker H3K27Ac. Immunoprecipitation with anti-H3K27Ac Ab significantly enriched for CCNE1 and CCNE2 (Supplementary Figure 5B), confirming CCNE1 and CCNE2 is a direct E2F8 target. Collectively, these results demonstrate that E2F8 can upregulate cyclin E1 and cyclin E2 in breast cancer by binding to the promoter of the CCNE1 and CCNE2 genes to activate their transcription.

### Clinical relevance of E2F8-induced cyclin E1 and cyclin E2 in human breast cancer

**Figure 7:**
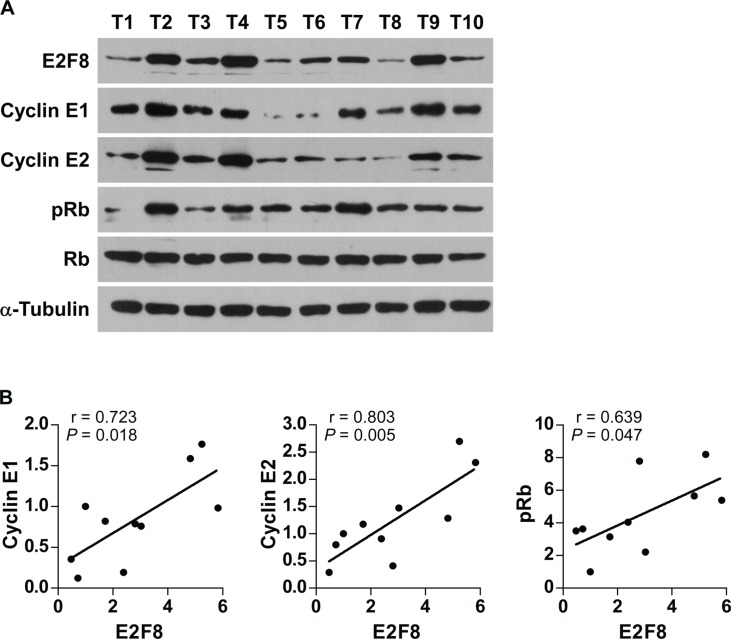
Relevance of E2F8-induced cyclin E1 and cyclin E2 activation in human cancers (**A**) Western blotting assay showing that the protein expression of E2F8, cyclin E1, cyclin E2, p-Rb and total Rb in 10 breast cancer samples. α-Tubulin was used as the loading controls. (**B**) Correlation analysis of E2F8 and cyclin E1, cyclin E2 and p-Rb expression, respectively. The expression levels of E2F8, Cyclin E1 and Cyclin E2 were determined by densitometry. The ratio of first sample (E2F8/α-tubulin, Cyclin E1/α-tubulin, Cyclin E2/α-tubulin) was considered as 1.0. r, Pearson correlation coefficient.

Finally, we examined whether E2F8-mediated cyclin E1 and cyclin E2 activation in breast cancer cells was clinically relevant. As shown in Figure 7A and 7B, E2F8 levels in 10 freshly collected breast cancer samples were significantly positively correlated with levels of cyclin E1 (*r* = 0.723, *P* = 0.018), cyclin E2 (*r* = 0.803, *P* = 0.005), and phosphorylation level of Rb (*r* = 0.639, *P* = 0.047). Collectively, these results further support the notion that upregulation of E2F8 contributes to uncontrolled cell proliferation and tumorigenecity, resulting in poor clinical outcome in breast cancer.

## DISCUSSION

E2F proteins have been proved to be important regulators of many processes relevant to cancer. For instance, the most studied member E2F1 maintained centrosome amplification and inhibited the promoter activity of the tumor suppressor gene ARHI, contributing to the tumorigenesis of breast cancer [18, 19]. Newly identified E2F8 acts as a potent cell cycle regulator, and has been emerging as a critical proliferation promoter in several human cancers [28–30]. However, the clinical significance and biological role of E2F8 in breast cancer remain largely unknown. This study establishes a vital role for E2F8 as a promoter of breast cancer proliferation and tumorigenecity. Significantly, we found that E2F8 was upregulated and correlated with clinical progression and poor prognosis in human breast cancer. Furthermore, our results reveal a potential molecular mechanism by which E2F8 promotes cell proliferation and tumorigenicity in breast cancer, via transcriptionally activating the CCNE1, and CCNE2 promoters. Taken together, these findings provide strong evidence that upregulation of E2F8 plays important roles in promoting breast cancer progression, and E2F8 might represent a novel prognostic biomarker and therapeutic target for the disease.

Based on gene expression profiles and genomic characterization, four main breast cancer subtypes have been proposed: luminal A, luminal B, HER2-enriched and basal-like [35, 36]. Subtype information has been shown to be an independent predictor of survival in breast cancer, where the luminal A subtype has a favorable prognosis compared to other subtypes [37]. The luminal B, basal-like and Her2-enriched subtypes include a characteristic signature containing high expression of genes associated with cell proliferation whereas luminal A has a low proliferation rate; however the mechanism remains unclear [31]. Herein, we found that E2F8 was further elevated in luminal B, basal-like and Her2-enriched subtypes than luminal A. E2F8 expression levels positively correlated with the proliferation marker Ki67 in patient tissues and *in vivo* tumor models. Thus, our findings suggest that E2F8 contributes to the proliferation capabilities in different breast cancer subtypes.

The functions of E2F8 on cell cycle progression and tumorigenesis in breast cancer are still unclear. In the present work, overexpression of E2F8 induced the expression of multiple cell cycle regulators, and upregulated cylin E1 and cyclin E2 by directly targeting their promoter elements, leading to G1/S phase transition and high cell proliferation rate. The CDK2-cyclin E complex was well known as they play an important role in tumor development through regulation of the cell cycle [38–40]. The expression of cyclin E1 (CCNE1) and cyclin E2 (CCNE2) was found to be strongly activated by the E2F transcription factors, such as E2F1 [41, 42], and the CDK2-cyclin E complex phosphorylated and inactivated Rb, while the phosphorylated Rb released E2F transcription factors, thereby promoting cell-cycle progression from G1 to S phase led to tumor regressions [43–45]. Thus, it would be of great interest and importance to investigate whether E2F8 upregulates cyclin E1, and cyclin E2 expression induce proliferation and tumorigenesis in breast cancer via Rb-E2F pathway which is critical in regulating in initiation of DNA replication.

The E2F proteins are conventionally considered to function as either activators (E2F1-3a) or repressors (E2F3b-8) of transcription during cell cycle regulation [46]. E2F8 was initially found to act as a repressor, as downregulated E2F-target genes blocked cell-cycle progression in fibroblasts [47, 48]. However, emerging evidence has revealed that E2F family members can function as either transcription activators or repressors, depending on the cellular and tissue context, or target genes [49–52]. Interestingly, E2F8 stimulate transcriptional activation of VEGFA in hypoxic cells, and on the other hand E2F8 repressed transcription of VEGFR1/2 in endothelial cells [25, 53]. E2F8 directly stimulate the CCBE1 promoter activity, but silence the promoter of FLT4 to control lymphangiogenesis during zebrafish embryonic development [26]. In addition, E2F8 was reported to transcriptionally upregulate cyclin D1 in hepatocellular carcinoma [29], and UHRF1 in lung cancer [30]. Thus, these recent advances indicate that E2F8 regulates a variety of downstream genes in a context-dependent manner. Herein, we report that E2F8 upregulates cyclin E1 and cyclin E2 expression by directly binding to these genes promoters, further supporting the notion that E2F8 promotes breast cancer proliferation and tumorigenicity by upregulating multiple cell cycle regulators. Considering that E2F8 is identified to activate the VEGFA promoter through cooperation with other transcriptional activator HIF1 [25], or the cyclin D1 promoter by blocking the occupancy of other E2Fs [29], it would be of great interest to investigate whether E2F8 may regulate CCNE1, and CCNE2 gene expression in breast cancer by either cooperating with other transcriptional activators or blocking the occupancy of other E2Fs.

In summary, our study has revealed that E2F8 upregulation plays an important role in breast cancer progression and E2F8 is a critical cell cycle promoter by directly upregulating CCNE1, and CCNE2. Understanding the precise role of E2F8 in breast cancer pathogenesis and in the cell cycle regulation promises to increase our knowledge of the biological basis of cancer development and may also facilitate the development of new therapeutic strategies against breast cancer.

## MATERIALS AND METHODS

### Cell lines and cell culture

Primary normal breast epithelial cells (NBEC1 and NBEC2) were established from the mammoplasty materials of two women at the Department of Plastic Surgery, the First Affiliated Hospital of Sun Yat-sen University, according to the rules and regulations relating ethical issues on research use of human subjects in China, as described previously [54]. Breast cancer cell lines (ZR-75-30, MCF7, BT549, DA-MB-453, MDA-MB-415, MDA-MB-231, MDA-MB-468, BT474, MDA-MB-361, SK-BR-3, and ZR-75-1) were obtained from American Type Culture Collection (ATCC) and cultured in the Dulbecco's Modified Eagle Medium (DMEM) (Invitrogen) with 10% fetal bovine serum (HyClone) at 37°C in a 5% CO_2_ atmosphere.

### Patients and tissue specimens

A total of 187 paraffin-embedded breast cancer specimens were collected for this study, which had been diagnosed histopathologically at the Sun Yat-sen University Cancer Center from 2002 to 2007. Clinical information of the samples is described in detail in Supplementary Table 1. The fresh tissues including eight paired breast cancer tissues and adjacent non-tumor tissues and ten breast cancer tissues were obtained from individuals who were diagnosed with breast cancer. Prior patient's consents and approval from the Institutional Research Ethics Committee were obtained to use these clinical specimens for research purposes.

### Western blotting analysis

Western blotting analysis was performed as described previously [54]. Briefly, cell were harvested in lysis buffer [25 mmol/L Tris (pH 6.8), 1% SDS, 5 mmol/L EDTA, protease inhibitor cocktail (Sigma)]. Protein concentration was determined with the bicinchoninic acid (BCA) assay (Pierce, Rockford, USA) according to the manufacturer's instructions. An anti-E2F8 mouse monoclonal antibody (1:1000 dilution; Abnova), an anti-cyclin E1 mouse monoclonal antibody (1:1000 dilution; Proteintech), an anti-cyclin E2 mouse monoclonal antibody (1:1000 dilution; Proteintech), an anti-Rb mouse monoclonal antibody (1:2000 dilution; Cell Signaling Technology), an anti-p-Rb rabbit polyclonal antibody (1:1000 dilution; Cell Signaling Technology), an anti-α-tubulin mouse monoclonal antibody (1:5000 dilution; Sigma) and the second antibody, goat anti-mouse immunoglobulin G (1:2000 dilution; Pierce), were used in this study.

### Immunohistochemistry

Immunohistochemistry (IHC) analysis was performed on the 187 paraffin-embedded breast cancer tissue sections as previously described [55]. The degree of immunostaining of formalin-fixed, paraffin-embedded sections were reviewed and scored separately by two independent pathologists. The scores were determined by combining the proportion of positively-stained cells and the intensity of staining. Cell proportions were scored as follows: 0, no positive cells; 1, < 10% positive cells; 2, 10%–35% positive cells; 3, 35%–75% positive cells; 4, > 75% positive cells. Staining intensity was graded according to the following standard: 1, no staining; 2, weak staining (light yellow); 3, moderate staining (yellow brown); 4, strong staining (brown). The staining index (SI) was calculated as the product of the staining intensity score and the proportion of positive cells. Using this method of assessment, we evaluated protein expression by determining the SI, with possible scores of 0, 2, 3, 4, 6, 8, 9, 12, and 16. Samples with a SI ≥ 8 were determined as high expression and samples with a SI < 8 were determined as low expression. Cutoff values were determined on the basis of a measure of heterogeneity using the log-rank test with respect to overall survival.

The method of MOD was used to determine the immunostaining intensity of each tested specimen and performed as previously reported [56]. Briefly, the stained sections were evaluated at ×200 magnification, and 10 representative staining fields of each section were analyzed to verify the MOD, which represents the strength of staining signals as measured per positive pixels. The MOD data were statistically analyzed using *t*-test to compare the average MOD difference between different groups of tissues, and *P* < 0.05 was considered significant.

### Plasmids, virus constructs and retroviral infection of target cells

Human CCNE1 and CCNE2 promoters were cloned, respectively, into the pGL3 luciferase reporter plasmid (Promega, Madison, WI, USA). Transfection of luciferase reporter plasmids was performed using the Lipofectamine 3000 reagent (Invitrogen) according to the manufacturer's instruction. Human E2F8 cDNA was PCR-amplified and cloned into the pMSCV-puro-retro vector (Clontech). Two short-hairpin RNA (shRNA) against E2F8 in pLKO-puro vector were commercially purchased (Sigma-Aldrich). Luciferase cDNA was PCR-amplified and cloned into the pMSCV-neo-retro vector (Clontech). Cells (2 × 10^5^) were seeded and infected by retroviral generated by pMSCV-puro-E2F8 or pLKO-puro-E2F8-shRNA transfecting in 293FT for 3 days. All these cells were further transfected with pMSCV-neo-luci plasmid. The stable cell lines expressing E2F8-luci and E2F8-shRNA-luci were elected with 0.5 μg/mL puromycin and 250 μg/mL G418 for 10 days. The sequences of primers were provided in the Supplementary Materials and Methods.

### MTT assay

Cell viability was measured using the 3-(4, 5-Dimethyl-2-thiazolyl)-2, 5-diphenyl-2H-tetrazolium bromide (MTT) assay as described previously [57]. 2 × 10^3^ cells were seeded per well in 96-well plates. At each time point, cells were dyed with 100 μL 0.5 mg/mL MTT for 4 hours at 37°C, followed by removal of the culture medium and addition of 100 μL of dimethyl sulphoxide. The absorbance was measured at 570 nm, with 655 nm as the reference wavelength.

### Anchorage-independent growth ability assay

Cells were trypsinized and suspended in 2 mL of complete medium plus 0.3% agar (Sigma) on 6-well plate (5 × 10^3^ cells per well). The cell mixture was plated on top of a bottom layer with 0.66% agar completed medium mixture. At 10 days, viable colonies that were larger than 0.1 mm were counted.

### Xenografted tumor model, IHC, and H & E staining

Female BALB/c-nu mice (5–6 weeks of age, 18–20 g) were purchased from the Center of Experimental Animal of Guangzhou University of Chinese Medicine, and were housed in barrier facilities on a 12 h light/dark cycle. All experimental procedures were approved by the Institutional Animal Care and Use Committee of Sun Yat-sen University. The mice were randomly divided into two groups (*n* = 6/group). One group of mice was inoculated subcutaneously with SK-BR-3/pMSCV-vector cells (5 × 10^6^) in the left mammary fat pad and with SK-BR-3/E2F8 cells (5 × 10^6^) in the right mammary fat pad per mouse. The other group of mice was inoculated subcutaneously with SK-BR-3/pSUPER-vector cells (5 × 10^6^) in the left mammary fat pad and with SK-BR-3/E2F8-sh#1 cells (5 × 10^6^) in the right mammary fat pad per mouse. 7 days later, kinetics of tumor formation was estimated by measuring tumor size at every 3 day interval. Tumor volume was calculated using the equation (L*W^2^)/2. On day 34, tumors were detected by an IVIS imagining system (Caliper), then animals were euthanized, tumors were excised, weighed and paraffin-embedded. Serial 6.0 μm sections were cut and subjected to immunohistochemical and H&E staining. After deparaffinization, sections were IHC analyzed using an anti-Ki67 (Dako, Glostrup, Denmark) or H & E stained with Mayer's hematoxylin solution. Proliferation index was quantized by counting proportion of Ki67-positive cells among the total number of invasive cells in the area scored based on the recommendations from the International Ki67 in Breast Cancer working group [58].

### Luciferase activity assays

Cells (3,000) were cultured in triplicate in 48-well plates for 24 h. 100 ng luciferase reporter plasmids or the control-luciferase plasmid, plus 1 ng pRL-TK Renilla plasmid (Promega), were transfected into cells using the Lipofectamine 3000 reagent (Invitrogen), according to the manufacturer's recommendations. Luciferase and Renilla signals were measured 24 h after transfection, using the Dual Luciferase Reporter Assay Kit (Promega), according to a protocol provided by the manufacturer.

### Chromatin immunoprecipitation

Cells (2 × 10^6^) in a 100 mm culture dish were treated with 1% final concentration of formaldehyde to cross-link proteins to DNA, and the reaction was stopped by addition of glycine. The cell lysates were sonicated to shear DNA to sizes of 300–1000 bp. Equal aliquots of chromatin supernatants were incubated with 1 μg of anti-E2F8, anti-H3K27Ac (Sigma) or anti-immunoglobulin G antibodies (Millipore, Billerica, MA, USA) overnight at 4°C with rotation. After reverse cross-link of protein/DNA complexes to free DNA, PCR was performed. Specific primers for chromatin immunoprecipitation (ChIP) were presented in the Supplementary Materials and Methods.

### Statistical analysis

All statistical analyses were carried out using the SPSS 17.0 statistical software package. The relationship between E2F8 expression and the clinicopathological characteristics was tested by the χ^2^ test. Bivariate correlations between study variables were calculated by Spearman's rank correlation coefficients. Survival curves were plotted with the Kaplan-Meier method and compared by the log-rank test. Survival data were evaluated using univariate and multivariate Cox-regression analyses. *P* < 0.05 in all cases was considered statistically significant.

## SUPPLEMENTARY MATERIALS TABLES AND FIGURES


