# Supplementary material for: Upregulation of E2F8 promotes cell proliferation and tumorigenicity in breast cancer by modulating G1/S phase transition
Source: Oncotarget. 2016 Mar 16;7(17):23757–71. doi: 10.18632/oncotarget.8121 (PMC5029661; doi:10.18632/oncotarget.8121)
Supplement: Supplementary file 1 [file oncotarget-07-23757-s001.pdf]

Upregulation of E2F8 promotes cell proliferation and tumorigenicity in breast cancer by modulating G1/S phase transition

Supplementary Materials

SUPPLEMENTARY MATERIALS AND METHODS

RNA extraction, reverse transcription (RT) and real-time PCR

Total RNA was extracted from cultured cells using the Trizol reagent (Invitrogen, Carlsbad, CA, USA) according to the manufacturer's instructions. 2 μg extracted RNA from each sample was used for cDNA synthesis with M-MLV Reverse Transcriptase (Promega, Madison, US). cDNAs were amplified and quantified by SYBR-Green in ABI Prism 7500 Sequence Detection System (Applied Biosystems, Texas, US). Expression data were normalized to the housekeeping gene GAPDH and calculated as $2^{-[(\text{Ct of } gene) - (\text{Ct of } GAPDH)]}$, where Ct represents the threshold cycle for each transcript.

Flow cytometry

Cells were harvested and fixed in 75% ethanol, and stored at 4°C overnight for later cell cycle analysis using flow cytometry. The fixed cells were centrifuged at 1,000 rpm for 5 minutes and washed with cold 1 × PBS twice. RNase A (20 μg/ml final concentration) and propidium iodide staining solution (50 μg/mL final concentration) were added to the cells and incubated for 30 minutes at 37°C in the dark. 2×10^5 cells were analyzed using a FACSC alibur instrument (BD Biosciences) equipped with CellQuest 3.3 software. Modfit LT 3.1 trial cell cycle analysis software was used to determine the percentage of cells in the different phases of the cell cycle.

Bromodeoxyuridine incorporation assay

To evaluate the cell population in S phase of the cell cycle, cells were incubated with bromodeoxyuridine (BrdU; Sigma-Aldrich) for 1 h and incubated with anti-BrdU antibody (Upstate, Billerica, MA, USA). The samples images were acquired under a laser scanning microscope (Axioskop 2 plus; Carl Zeiss Co Ltd, Oberkochen, Germany).

Transwell assay

Cells (2×10^4) were seeded on the transwell inserts (pre-coated with Matrigel for invasion assay; didn't pre-coat with Matrigel for migration assay) with 8 μm micropore filters (Corning Costar) in 500 μl medium. Medium containing 10% FBS was added to the lower chamber as a chemoattractant. After 24 h, cells on the upper side of the filter were removed with a cotton swab. Cells that had penetrated to the lower membrane surface were fixed in 1% paraformaldehyde, stained with hematoxylin and counted under an optical microscope (×100 magnification). Cell counts are expressed as the mean number of cells from 10 random fields per well.

Primers and oligonucleotides:

**Real-time PCR primers:**		
Gene	Forward primer (5′–3′)	Reverse primer (5′–3′)
E2F8	GAAATCCCAACCAAGTCGAA	CTTCGTCAAGGCAGATGTCA
CCNE1	CGGTATATGGCGACACAAGA	ACATACGCAAACTGGTGCAA
CCNE2	AGGAAAACTACCCAGGATGTCA	ATCAGGCAAAGGTGAAGGATTA
GAPDH	GCACCGTCAAGGCTGAGAAC	TGGTGAAGACGCCAGTGGA
**The primers used for promoter luciferase reporter:**		
Gene	Forward primer (5′–3′)	Reverse primer (5′–3′)
CCNE1 promoter	AGGTACCGAGCTCTTACGCGTGCCATG ATACTTTGAAGGACTTAGCCC	TGCAGTCGGGGCGGCAGATCTCAGGAGT CCCTGTCCGCAG
CCNE2 promoter	AGGTACCGAGCTCTTACGCGTGCCCC AGTCTCCTTTCCCTCCTTC	TGCAGTCGGGGCGGCAGATCTGCATT CTGTTCACATAATAGTCAAGG
**The primers used for ChIP:**		
Gene	Forward primer (5′–3′)	Reverse primer (5′–3′)
CCNE1 p1	ATGATACTTTGAAGGACTTAGCCC	GCCCTTCTTAAGCTAAAGTCTCAG
CCNE1 p2	GGACAGGACTGAGACTTTAGCTTAA	CTTGTCTCTCTCCCCACCTTG
CCNE1 p3	CAGTGAGCAAGATGGGCAAG	ACAGGACCTGACCCTGATCC
CCNE1 p4	GGAGAGGAGGCCTGAGGTC	TGGCTCTCTGAAGACCTTTCTG
CCNE1 p5	GGAATCCCAGAGTCAGAAAGGT	GGACATCCCCAAGGTCACC
CCNE1 p6	CGGTGACCTTGGGGATGT	GGACGCGGGAGAAGTCTG
CCNE1 p7	GCCAGACTTCTCCCGCGT	GGCTCAGAGCGGGACATTTA
CCNE1 p8	TAAATGTCCCGCTCTGAGCC	CAGGAGTCCCTGTCCGCAG
CCNE2 p1	CCAGTCTCCTTTCCCTCCTTC	GAGAGCCGCCCCTCATTC
CCNE2 p2	CCAAATGAGGGTGGGATAGAG	GTCTGGCGGTGAGGAGTTG
CCNE2 p3	CGCAGCAACTCCTCACCG	GAGAGTTTCCCTACCGCCG
CCNE2 p4	ACTCTACCGGGCCTTCTGC	GAAAGACCTGGGTTCCCTGAG
CCNE2 p5	CCTAGCTGTCCCGCCAAG	GGAAAGCAGGGTTGATACATACC
CCNE2 p6	GGTATGTATCAACCCTGCTTTCC	CGTGGTGGCGATCTTTCTTC
CCNE2 p7	CGGGTGGGAAGAAAGATCG	AAAACCTGTTTGCGGAATACC
CCNE2 p8	AACTGCACATCGTCAAGTCAGAC	AAGTGTGAAAAAGGACTTCGCC
CCNE2 p9	GCGGGCGAAGTCCTTTTT	CCTGACCCCTTTTCTTGACATC
CCNE2 p10	AAAGGTCTAACAGCATGATGTCAAG	GCATTCTGTTCACATAATAGTCAAGG

Supplementary Table S1: Clinicopathological characteristics of studied patients and expression of E2F8 in breast cancer

Characteristics		Total	(%)
Age (years)	< 48	91	48.7
	≥ 48	96	51.3
Gender	Male	0	0.0
	Female	187	100.0
Clinical stage	I	33	17.6
	II	95	50.8
	III	51	27.3
	IV	8	4.3
T classification	T1	55	29.4
	T2	103	55.1
	T3	18	9.6
	T4	11	5.9
N classification	N0	84	44.9
	N1	54	28.9
	N2	32	17.1
	N3	17	9.1
M classification	M0	180	96.3
	M1	7	3.7
Estrogen Receptor (ER)	0	87	46.5
	1	35	18.7
	2	18	9.6
	3	46	24.6
	4	1	0.5
Progesterone Receptor (PR)	0	83	44.4
	1	33	17.6
	2	34	18.2
	3	37	19.8
Erb-b2 receptor tyrosine kinase 2 (ErbB2)	0	92	49.2
	1	27	14.4
	2	22	11.8
	3	46	24.6
Ki67	0	34	18.2
	1	69	36.9
	2	52	27.8
	3	32	17.1
E2F8 expression	Low	95	50.8
	High	92	49.2
Vital status	Alive	131	70.1
	Dead	56	29.9

Supplementary Table S2: Correlation between the clinicopathological features and expression of E2F8

Characteristics		E2F8		X^2 test p value
		Low expression (50.8%)	High expression (49.2%)	
Age (years)	< 48	48	43	0.604
	≥ 48	47	49	
Clinical stage	I	21	12	0.007
	II	54	41	
	III	19	32	
	IV	1	7	
T classification	T1	33	22	0.031
	T2	54	49	
	T3	6	12	
	T4	2	9	
N classification	N0	53	31	0.014
	N1	25	29	
	N2	11	21	
	N3	6	11	
M classification	M0	94	86	0.049
	M1	1	6	
ER	0	39	48	0.290
	1	17	18	
	2	11	7	
	3	28	18	
	4	0	1	
PR	0	42	41	0.921
	1	16	17	
	2	19	15	
	3	18	19	
ErbB2	0	49	43	0.926
	1	13	14	
	2	11	11	
	3	22	24	
Ki67	0	20	14	0.005
	1	44	25	
	2	17	35	
	3	14	18	
Vital status	Alive	80	51	< 0.001
	Dead	15	41	

Supplementary Table S3: Univariate and multivariate analysis of different prognostic parameters in patients with breast cancer by Cox-regression analysis

	Univariate analysis		Multivariate analysis	
	P	**Hazard ratio (95% CI)**	***P***	**Hazard ratio (95% CI)**
**Clinical stage**				
I-II	< 0.001	2.864 (1.693–4.845)	0.001	2.424 (1.416–4.150)
III-IV				
**E2F8**				
Low expression	< 0.001	3.619 (1.999–6.553)	0.001	2.697 (1.463–4.974)
High expression				
**Ki67**				
Low expression	0.006	2.137 (1.249–3.656)	0.022	1.896 (1.099–3.271)
High expression				

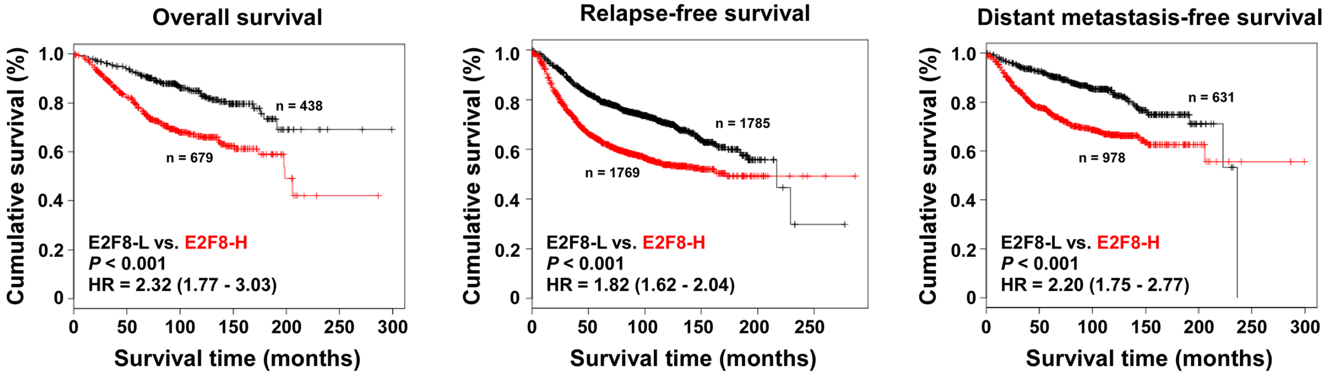

Supplementary Figure S1: Kaplan-Meier survival curves from publicly available microarray data for breast cancer patients. Overall survival, relapse-free survival and distant metastasis-free survival in breast cancer patients with high and low levels of E2F8 mRNA (auto selecting best cutoff) were analyzed using the publicly accessible tool KM Plotter (http://kmplot.com/breast/). The two cohorts of patients were compared by log-rank tests, and the hazard ratio with 95% confidence intervals was calculated.

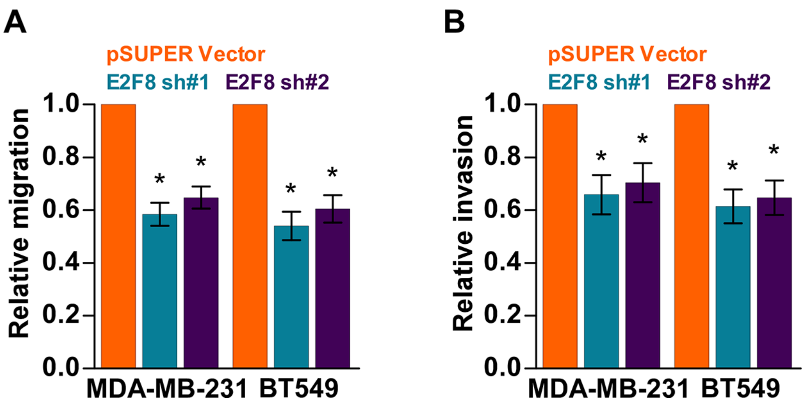

Supplementary Figure S2: Effect of E2F8 overexpression and knockdown on cell migration and invasion. (**A**) Transwell migration assays. The cells were transferred into transwell inserts without matrigel-coated membranes and assessed 24 hours after incubation. (**B**) Transwell invasion assays. The cells were transferred into transwell inserts containing matrigel-coated membranes and assessed 24 hours after incubation. Mean ± SD in three independent experiments. Two sided *t* test. $^*P < .05$.

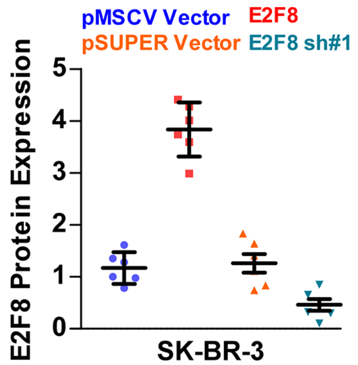

Supplementary Figure S3: Expression of E2F8 in xenografts examined by western blot. E2F8 was robustly upregulated in tumors formed by SK-BR-3/E2F8 cells, but downregulated in tumors formed by E2F8-silencing SK-BR-3 cells.

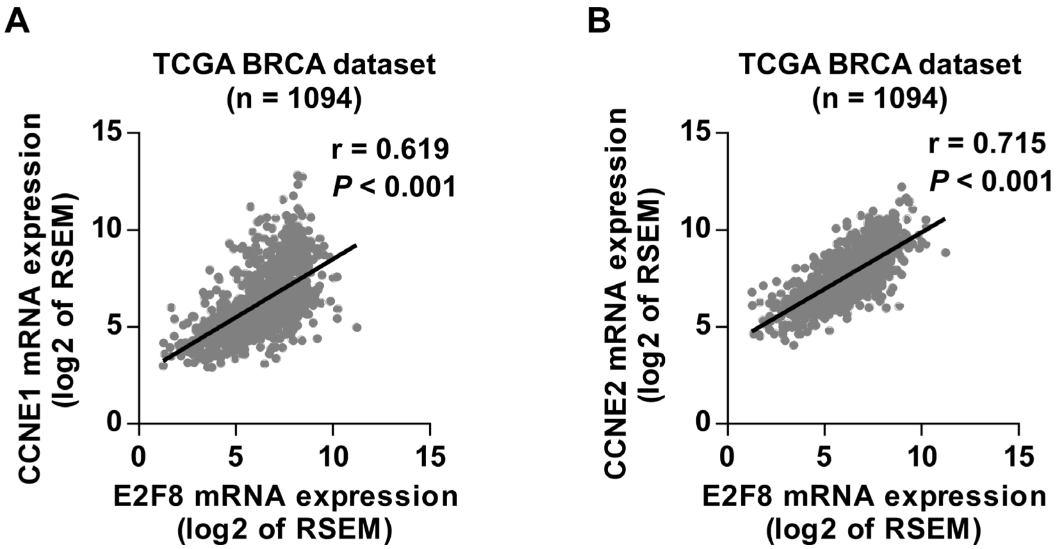

Supplementary Figure S4: Expression of E2F8 positively correlating with CCNE1 and CCNE2. Bivariate correlations between E2F8 and CCNE1(**A**)/CCNE2 (**B**) mRNA expression by Spearman's rank correlation coefficients from TCGA BRCA mRNA data set (n = 1094).

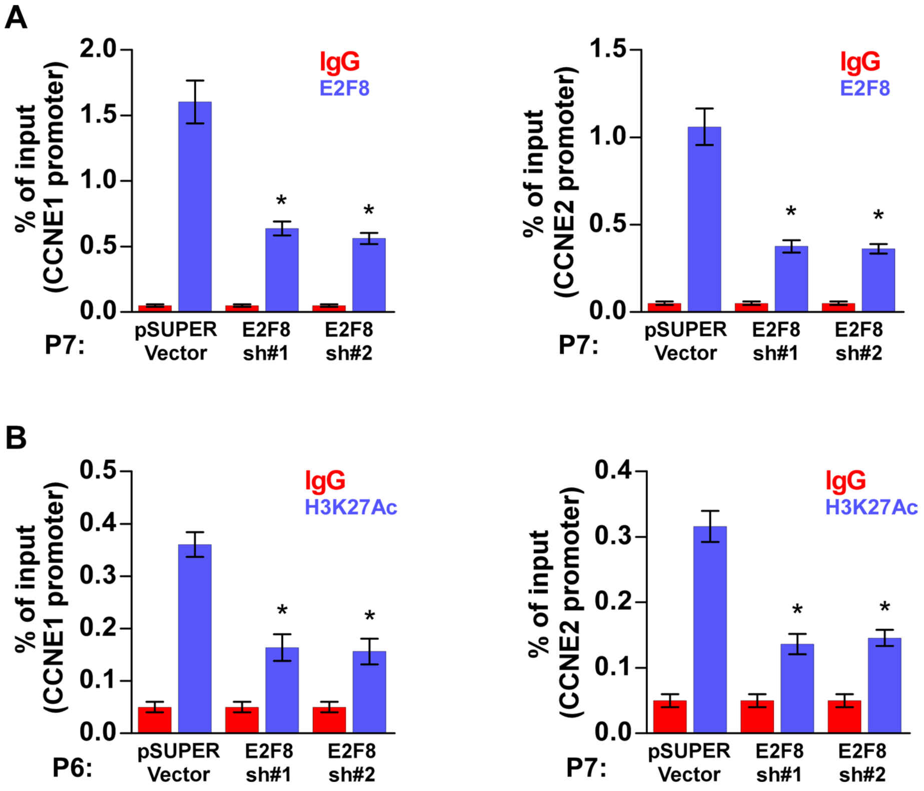

Supplementary Figure S5: ChIP-validated E2F8 binding to CCNE1 and CCNE2 promoter. (**A**) ChIP enrichment assay confirms that E2F8 binds to both the P7 ChIP primer PCR site of CCNE1 and CCNE2 in the E2F8-silencing cell. (**B**) The P6 ChIP primer PCR site of CCNE1 and the P7 ChIP primer PCR site of CCNE2 promoters enriched by pulling downing transcription activation marker H3K27Ac or IgG. IgG was used as a negative control. Results were evaluated from three independent experiments, $^*P < 0.05$.
